# Exogenous Semaphorin 3E treatment protects against chlamydial lung infection in mice

**DOI:** 10.3389/fimmu.2022.882412

**Published:** 2022-08-02

**Authors:** Rony Thomas, Shuhe Wang, Rasheduzzaman Rashu, Ying Peng, Abdelilah S. Gounni, Xi Yang

**Affiliations:** Department of Immunology, University of Manitoba, Winnipeg, MB, Canada

**Keywords:** *Chlamydia muridarum*, Semaphorin 3E, Th1/Th17 cells, Tregs, dendritic cells

## Abstract

Recent studies reported that semaphorins play a significant role in various settings of the immune response. In particular, Semaphorin 3E (Sema3E), a secreted semaphorin protein, is involved in cell proliferation, migration, inflammatory responses, and host defence against infections. However, the therapeutic function of Sema3E in bacterial infection has not been investigated. Our data showed that exogenous Sema3E treatment protects mice from chlamydial infection with lower bacterial burden, reduced body weight loss, and pathological lung changes. Cytokine analysis in the lung and spleen revealed that Sema3E-Fc treated mice, compared to saline-Fc treated mice, showed enhanced production of IFN-γ and IL-17 but reduced IL-4 and IL-10 production. Cellular analysis showed that Sema3E treatment leads to enhanced Th1/Th17 response but reduced Treg response in lungs following chlamydial infection. Moreover, Sema3E treatment also enhanced the recruitment of pulmonary dendritic cells, which express higher co-stimulatory but lower inhibitory surface molecules. The data demonstrate that Sema3E plays a vital role in protective immunity against chlamydial lung infection, mainly through coordinating functions of T cells and DCs.

## Introduction

*Chlamydiae* are gram-negative obligate intracellular bacterium (1). Two major species of *Chlamydia* that infects human are *Chlamydia pneumonia* and *Chlamydia trachomatis* (2–4). Mouse infection with *Chlamydia muridarum* (*Cm*) has been commonly used to study the immunobiology of respiratory and genital tract chlamydial infections. Studies in recent years have elucidated the critical role of cell-mediated immunity in the protective immune response to chlamydial infection. Previous studies by us and others have suggested that IFN-γ production by T cells plays a major function in resolving chlamydial infection (5–7). In addition, IL-17 production by T cells is also found to protect against chlamydial infection in the lungs (7–9). In contrast, Th2 and regulatory T cells (Tregs) response lead to an immunopathological response to chlamydial infections (10, 11). Many studies documented the incredible ability of dendritic cells to influence T cell response to chlamydial infection. Surface expression of molecules such as CD40, CD80, and CD86 provide co-stimulation to enhance T cell response (12). The expression of inhibitory molecules, such as programmed cell death ligand-1 (PD-L1) by dendritic cells (DCs), reduces Th1/Th17 response to chlamydial infection (13).

Semaphorins are a large family of proteins described initially as axon guidance cues involved in neural development (14). Studies over the past decade indicated the critical role of semaphorins in cell proliferation, tumour growth, angiogenesis and immune functions (15). Semaphorin 3E (Sema3E) is a secreted protein that plays diverse functions in immune cells in a context-dependent manner (16–18). Sema3E acts as a chemoattractant for macrophages in adipose tissue, and p53-induced upregulation of Sema3E leads to tissue inflammation (19). On the other hand, Sema3E is expressed in macrophages of atherosclerotic plaques and inhibits macrophage migration to chemokine by disrupting the re-organization of the actin cytoskeleton (20). Sema3E is expressed in numerous cell types such as adipocytes (19), DCs (21), thymic epithelial cells (22), macrophages (20), tumour cells (17), and osteoblasts (23). Sema3E binds to the receptor PlexinD1(PlxnD1), which is highly expressed in embryonic tissues, osteoblasts, lung mesenchyme, adrenal gland, mammary gland, small intestine, and immune cells (24). Sema3E deficiency in mice leads to exaggerated allergic airway inflammation, remodelling, and airway hyperresponsiveness, while intranasal recombinant Sema3E treatment reduced house dust mite-induced allergic asthma (25, 26). Intranasal exogenous Sema3E protects mice from allergic asthma by reducing eosinophilic inflammation, serum IgE, and Th2 cytokines (26). We recently reported that Sema3E played an important role in host defence against bacterial infection by showing that Sema3E-deficient mice exhibited more severe disease and higher bacterial growth following *Chlamydia muridarum* lung infection (27). In addition, we found that DCs from *Chlamydia*-infected Sema3E KO mice failed to induce protective T cell responses (27). The purpose of this study by delivering exogenous Sema3E to wild-type (WT) and Sema3E deficient mice was, on the one hand, to further test the conclusion using a complementary approach and, on the other hand, to test the therapeutic potential of Sema3E in promoting immunity against bacterial infections.

This study found that treatment of WT and Sema3E KO mice with recombinant Sema3E-Fc protected the mice from chlamydial infection with lower bacterial burden and inflammation in the lung. The protective effect was associated with enhanced Th1/Th17 response but reduced Treg response. The exogenous Sema3E treatment also increased the expression of co-stimulatory molecules and reduced the immune-inhibitory marker PD-L1 on the surface of DCs. The recruitment/expansion of DCs, including the CD103+ subset to the site of infection, was also increased. Altogether, the study verified the importance of Sema3E in host defence against bacterial infection and suggested that a supplement of Sema3E could be a potential strategy to enhance protection against bacterial infection or vaccination.

## Materials and methods

### Animals

Sema3E^-/-^ BALB/c mice were obtained from a GMC animal house at the University of Manitoba, Winnipeg, Canada. Sema3E^-/-^ mice in B6 background were gifted by Dr. F. Mann, Université de la Méditerranée, Marseille, France. These mice were backcrossed for ten generations to obtain Sema3E^-/-^ mice in BALB/c background. All mice were maintained in the Animal Care Facility of the University of Manitoba. The immunophenotypic analysis of Sema3E^*−/−*^ mice has been shown previously (25). The use of all mice in this study was in adherence to the ethical standards prescribed by the Canadian Council on Animal Care (CCAC) and The University of Manitoba Animal Ethics Committee (Protocol # 19-029).

### Organism

*Chlamydia muridarum* (*Cm*) used in this study was propagated and cultured, as described previously (28). Shortly, HeLa 229 cell monolayers in Eagle’s MEM (composed of 10% FBS and 2 mM L-glutamine) were infected with *Cm* for 48 h. Infected cells were harvested using sterile glass beads, and *Cm* elementary bodies (EBs) were isolated by discontinuous density gradient centrifugation. The purified *Cm* elementary bodies were stored in the sucrose-phosphate-glutamic acid buffer (SPG) at −80°C.

### Infection of mice and quantification of chlamydial *in vivo* growth

Mice were infected intranasally with 1×10^3^ inclusion-forming units (IFU) of *Cm* in 40 μl of SPG buffer. Infected mice were sacrificed on day 7 post-infection. Three mice were used per group in each experiment. The chlamydial load in the lung was determined as described previously (28). Briefly, lung tissue suspensions aseptically isolated from mice were homogenized using a cell grinder in SPG buffer. Homogenized tissue was centrifuged at 1900 × g for 30 min at 4°C, and the supernatant was collected and kept at −80°C. HeLa 229 cells were grown to confluence in 96-well flat-bottom microtiter plates for Cm quantitation. The monolayers were then washed in 100 μl of Hank’s Balanced Salt Solution (HBSS), inoculated in triplicates with 100 μl of serially diluted samples, and incubated at 37°C for 2 hours. After washing plates, 200 μl of MEM containing cycloheximide (1.5 μg/ml) and gentamicin (10 μg/ml) was added. The plates were incubated at 37°C in 5% CO2. After 48 hours, the culture medium was removed, and the cells were fixed with absolute methanol. Fixed cells were washed and incubated with *Chlamydia* genus-specific murine mAb at 37°C for 70 minutes. The cells were washed, stained with HRP conjugated goat anti-mouse IgG, and developed with a 4-chloro-1-naphthol (Sigma-Aldrich) substrate. The number of inclusions was counted under a microscope. Five fields per well were counted, and the chlamydial load was analyzed based on dilution titers of the original inoculum.

### Semaphorin 3E treatment of mice

Recombinant mouse Semaphorin 3E Fc protein (Sema3E-Fc) and control IgG Fc in saline (saline-Fc) were purchased from R&D SYSTEMS and used according to the manufacturer’s instructions. WT BALB/c mice and Sema3E KO BALB/c mice were treated intranasally with either Sema3E-Fc or saline-Fc (0.3 μg per mouse) two hours before *Cm* infection and day 1 to day 6 consecutively after *Cm* infection. Mice were sacrificed on day 7 post-infection and analyzed for bacterial load and cytokine response (**Supplementary Figure 1A**). To analyze the surface phenotype of DCs following *Cm* infection, Sema3E KO and WT mice were infected intranasally with *Cm* and treated with Sema3E-Fc, or saline-Fc, and sacrificed after 3 days.

### Isolation of lung and spleen for the preparation of single-cell suspensions

For obtaining single lung cell suspensions, lung tissues isolated from mice at designated time periods of infection were digested in 2 mg/ml collagenase XI (Sigma-Aldrich, Oakville, Ontario, Canada) dissolved in RPMI 1640 medium at 37°C for 60 min. In the last 5 min of incubation, EDTA (2 mM, pH 7.2) was also added to the medium. After filtering cells through 70 μm cell strainers, red blood cells (RBC) were lysed by ACK lysis buffer (composed of 150 mM NH_4_Cl, 10 mM KHCO_3_, and 0.1 mM EDTA). Spleen single-cells were made by digesting spleens with 2 mg/mL collagenase D (Roche Diagnostics, Meylan, France) in RPMI 1640 for 30 min at 37°C. The RBCs were then lyzed by using ACK lysis buffer. The isolated cells were washed and resuspended in complete RPMI-1640 medium (RPMI-1640 supplemented with 10% FBS, 1% L-glutamine, 25 mg/ml gentamicin) for further analysis.

### Quantification of cytokines

Single-cell suspensions were cultured with ultraviolet-killed elementary bodies (UVEB) (1 × 10^5^ IFU/ml) at a concentration of 7.5 × 10^6^ (for spleen) and 5 × 10^6^ (lung) cells/well. The supernatants were collected after 72 hours and assayed for the production of cytokines IFN-γ, IL-17, IL-10, and IL-4 (eBioscience or BD Biosciences) by enzyme-linked immunosorbent assay (ELISA).

### DC purification

Purification of DC was performed, as described previously (29). Briefly, splenic and lung single-cell suspensions were incubated with CD11c microbeads (Miltenyi Biotec) for 15 minutes at 4°C and passed through magnetic columns for positive selection. The purity of DC was found to be >95%.

### Flow cytometry

Surface marker expression on DC was analyzed by staining freshly isolated lung DC with anti-CD11c-APC, anti-MHC class II PE-Cy7, anti-CD40 FITC, anti-CD80 FITC, or anti-CD86 FITC, anti-PD-L1-PE, anti-F4/80-PE (eBioscience, San Diego, CA), or isotype controls in a flow staining buffer (composed of Dulbecco’s PBS mixed with 2% FBS as well as 0.09% NaN3). After surface staining, cells were fixed using 2% paraformaldehyde for 30 minutes, washed twice, and resuspended in staining buffer. Lung DCs were identified according to CD11c^hi^MHC-II^hi^F4/80^-^ expression (**Supplementary Figure 1B**).

For T cell intracellular cytokine analysis, lung single-cell suspensions were cultured at 7.5 × 10^6^ cells/well in the presence of phorbol 12-myristate 13-acetate (50 n g/ml; Sigma-Aldrich, St Louis, MO, USA) and ionomycin (1 μg/ml; Sigma-Aldrich) in complete RPMI 1640 medium for 6 h at 37°C. Brefeldin A (5 μg/ml; eBioscience) was added at the last 3 hours of incubation to accumulate cytokines intracellularly. Cultured cells were then washed twice and incubated with Fc receptors blocking Abs (anti-CD16/CD32 antibody; eBioscience) for 15 min on ice to prevent non-specific staining. Following this step, surface marker staining was done on cells using fluorescent-labelled anti-CD3 PE-Cy7, anti-CD4 FITC, anti-CD25 APC, or anti-CD8a PE mAbs (eBioscience). The surface-stained cells were then fixed and permeabilized using Cytofix/Cytoperm (BD Pharmingen). Later, cells were intracellularly stained with anti-IFN-γ-APC, anti-IL-17-APC, or isotype control antibodies (eBioscience). After staining, cells were then washed, resuspended in staining buffer, and data were collected by BD FACSCanto™ II (BD Biosciences) and analyzed using FlowJo. FoxP3 staining was done using Foxp3/Transcription Factor Staining Buffer Set (eBioscience) according to the manufacturer’s instructions and stained using anti-Foxp3-PE or isotype control antibodies (eBioscience). For CD4 and CD8 T cells, the analysis was performed on gated CD3^+^ cells. CD4^+^ FoxP3^+^CD25^+^ cells gated on CD4 T cells were analyzed as Tregs (**Supplementary Figure 1C**).

### Histopathological analysis

The lung tissues aseptically obtained from different mice groups at indicated time points were fixed in 10% formalin. Haematoxylin and Eosin (H&E) staining was done on tissue sections, and histopathological changes were observed under light microscopy, as described (7). The degree of lung inflammation was analyzed using a semi-quantitative grading system (9); grading scale: 0, normal; 1, mild inflammation, granuloma formation, cellular infiltration of less than 25% of the area, no prominent infiltration into adjacent alveolar septae or air space; 2, mild interstitial pneumonitis, diffused cellular infiltration in some region (25%–50%), septal congestion, interstitial edema; 3, inflammatory cell infiltration into perivascular, peribronchiolar, alveolar septae, and air space (50%–75% of the area); 4, over 75% of the area of the lung filled with infiltrating cells.

### Statistical analysis

Unpaired Student’s t-test (GraphPad Prism software v4, GraphPad, San Diego, USA) was used to assay the statistical significance of comparing two different groups. For comparing several groups of mice, a one-way analysis of variance (ANOVA) was used. A p-value of less than 0.05 was considered significant.

## Results

### Semaphorin 3E treatment protects against chlamydial lung infection

**Figure 1:**
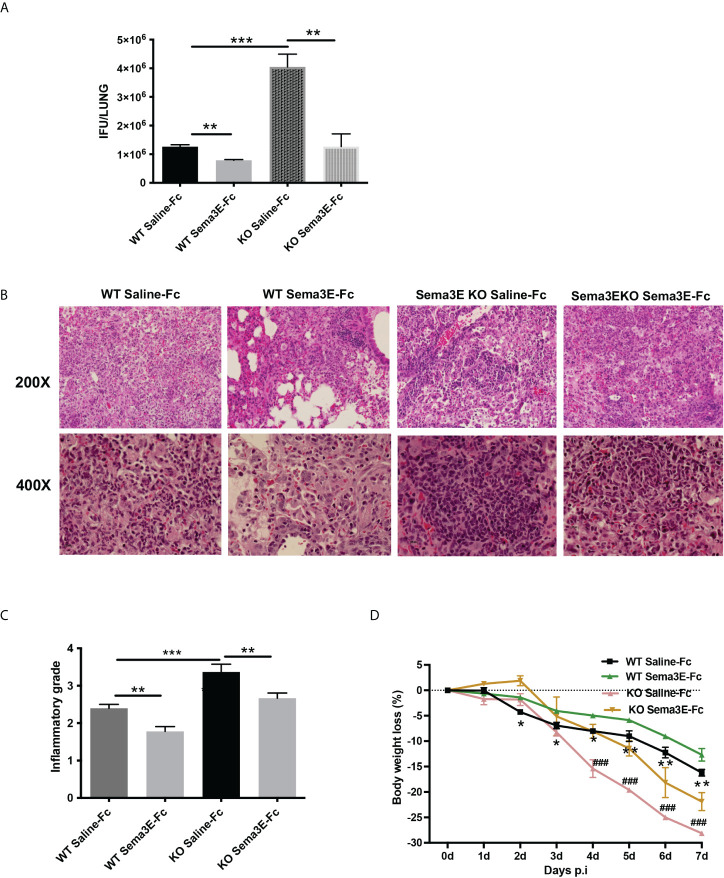
Semaphorin 3E treatment enhances the ability to control *C. muridarum* lung infection. Mice groups were treated with recombinant Sema3E-Fc intranasally 2 hours before *Cm* infection and day 1 to day 6 after infection. Mice were sacrificed at day 7 post-infection. **(A)** Chlamydial growth *in vivo* after Sema3E-Fc or saline-Fc treatment of WT and Sema3E KO mice. **(B)** The pulmonary pathology. Lungs from Sema3E-Fc or saline-treated mice were sectioned, and the lung pathology was examined by H&E staining and analyzed in (×200 and ×400x) magnification under light microscopy. **(C)** Lung inflammation was analyzed semi-quantitatively by a blinded pathologist as detailed in Materials and Methods. **(D)** Mice were monitored daily for body weight changes. After chlamydial infection, lower body weight loss was observed in Sema3E-Fc treated WT mice and Sema3E KO mice. Each point represents the mean ± SD of three mice. One representative experiment of three independent experiments with similar results is shown. Significant differences in the bodyweight loss of Sema3E-Fc treated and saline-Fc treated WT mice are shown as asterisks (*), differences in the bodyweight loss of saline-Fc treated WT mice and saline-Fc treated Sema3E KO mice are shown as hashtags (#). *p < 0.05, **p < 0.01, ***p < 0.001, **###** *p* < 0.001.

To evaluate the potential of exogenous Sema3E to enhance the capability to fight against chlamydial infection, we treated WT mice and Sema3E KO mice intranasally with either recombinant Sema3E-Fc or control saline-Fc, two hours before *Cm* infection and day 1 to day 6 consecutively after infection (**Supplementary Figure 1A**). In both WT and Sema3E KO mice, we observed a significant decrease in chlamydial load in the mice that received Sema3E-Fc compared to those that received saline-Fc alone (**Figure 1A**). In addition, lung histological analysis showed that mice treated with Sema3E-Fc exhibited a reduced inflammatory cell infiltration compared to saline-Fc–treated controls (**Figures 1B, C**). Similarly, it was found that Sema3E-Fc treatment reduced body weight loss in both WT and Sema3E KO mice compared to saline-Fc treated mice after *Cm* infection (**Figure 1D**). The observations that the supplement of exogenous Sema3E corrected the failure of KO mice in protection against infection and further enhanced the protection of WT mice confirm our previous finding on the crucial role of Sema3E in host defence against infection and the effectiveness of supplementing Sema3E to enhance immunity to infection further.

### Sema3E treatment promotes Th1/Th17 responses while reducing IL-4/IL-10 responses after chlamydial infection at the population level

**Figure 2:**
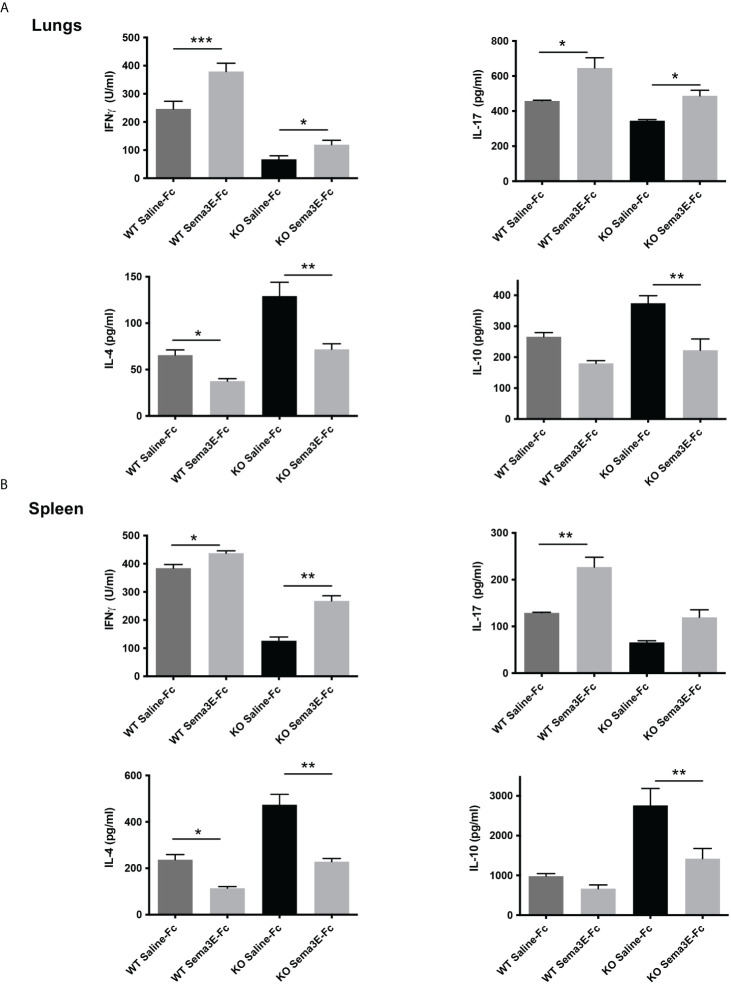
Semaphorin 3E treatment modulates antigen-driven cytokine responses after chlamydial infection. Exogenous recombinant Sema3E-Fc/Saline-Fc was administered to WT and Sema3E KO mice intranasally 2 hours before *Cm* infection (1x10^3^ IFUs of *Cm*) day 1 to day 6 after infection. Mice were sacrificed on day 7 post-infection. The spleen cells were cultured with UV-killed elementary bodies (UVEB). IFN-γ, IL-17, IL-10, and IL-4 levels in 72-h culture supernatants were determined by ELISA. **(A)** IFN-γ, IL-17, IL-10, and IL-4 cytokine production in lung cells at day 7 p.i. **(B)** IFN-γ, IL-17, IL-10, and IL-4 cytokine production in the spleen at day 7 p.i. Data are shown as mean ± SD (n = 3) and represent one of three independent experiments with similar results. *p < 0.05, **p < 0.01, ***p < 0.001.

To address the mechanism of Sema3E function in protection, we next investigated the effect of Sema3E-Fc treatment on cytokine response of mice after *Cm* lung infection. We first examined cytokine responses in the local lung tissues. Sema3E-Fc–treated WT and Sema3E KO mice showed a significant increase in IFN-γ and IL-17 cytokines in the lungs compared to those given control saline-Fc (**Figure 2A**). In contrast, IL-4 and IL-10 cytokines were reduced in Sema3E-Fc–treated mice (**Figure 2A**). To further understand the effect of Sema3E treatment on antigen-driven T cell immune response, we first studied cytokine responses by ex vivo splenocytes isolated from *Cm* infected mice upon UV killed *Cm* restimulation. Sema3E-Fc–treatment of WT mice, compared to saline-Fc treatment, increased antigen-driven IFN γ and IL-17 but reduced IL-4 production (**Figure 2B**). Similarly, Sema3E-Fc–treatment of KO mice also enhanced antigen-driven IFN γ but reduced IL-10 and IL-4 production. The results suggest that Sema3E can modulate cytokine responses differently by preferentially promoting Th1/Th17 while reducing IL-4/IL-10 responses after chlamydial infection.

### Sema3E treatment enhances Th1/Tc1 and Th17 cytokine response in the lung after chlamydial infection

**Figure 3:**
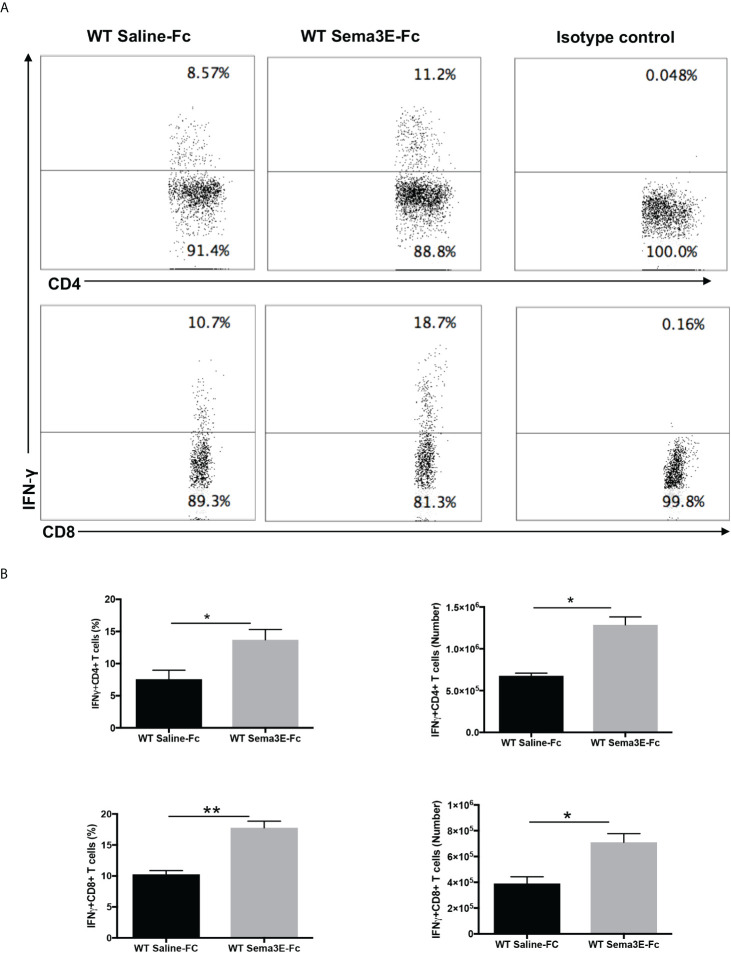
Higher IFN-γ production by CD4 and CD8 T cells after Sema3E-Fc treatment of *Cm* infected WT mice. CD4^+^ and CD8^+^ T cells isolated from the lungs of *Cm* infected mice at day 7 post-infection were stained intracellularly for IFN-γ. **(A)** Representative flow cytometric images and summary of flow cytometric analysis **(B)** to show the percentage and absolute number of IFN-γ producing CD4 and CD8 T cells. Data are shown as mean ± SD (n = 3) and represent one of three independent experiments with similar results. *p < 0.05, **p < 0.01.

**Figure 4:**
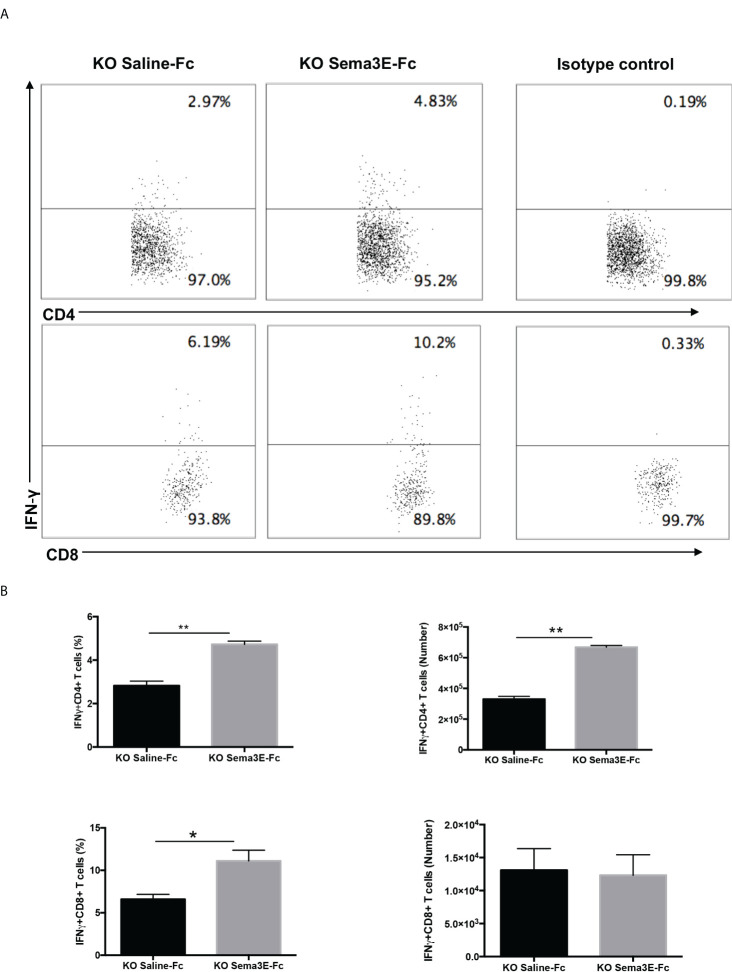
Higher IFN-γ production by CD4 and CD8 T cells after Sema3E-Fc treatment of *Cm* infected Sema3E KO mice. CD4^+^ and CD8^+^ T cells isolated from the lungs of *Cm* infected mice at day 7 post-infection were stained intracellularly for IFN-γ. **(A)** Representative flow cytometric images and summary of flow cytometric analysis **(B)** to show the percentage and absolute number of IFN-γ producing CD4 and CD8 T cells. Data are shown as mean ± SD (n = 3) and represent one of three independent experiments with similar results. *p < 0.05, **p < 0.01.

**Figure 5:**
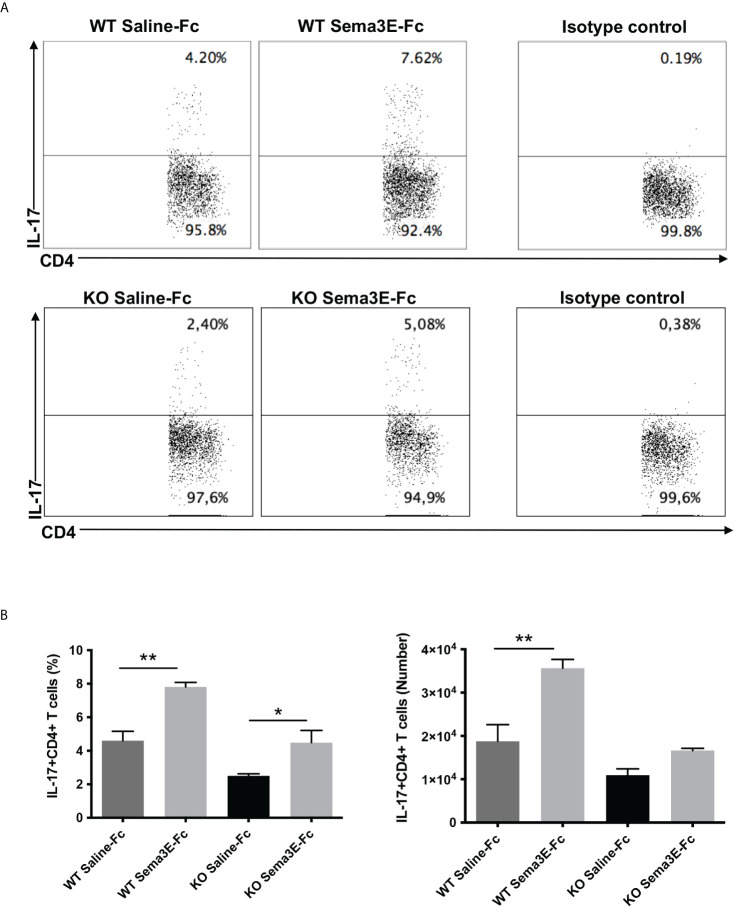
Higher IL-17 production by CD4 T cells after Sema3E-Fc treatment of *Cm* infected mice. CD4^+^ T cells isolated from the lungs of *Cm* infected WT and Sema3E KO mice at day 7 post-infection were stained intracellularly for IL-17. **(A)** Representative flow cytometric images and summary of flow cytometric analysis **(B)** to show the percentage and absolute number of IL-17 producing CD4^+^T cells. Data are shown as mean ± SD (n = 3) and represent one of three independent experiments with similar results. *p < 0.05, **p < 0.01.

We performed an intracellular cytokine analysis of lung CD4+ and CD8+ T cells by flow cytometry to analyze T cell cytokine responses at a single-cell level. The intracellular cytokine analysis showed a higher number of IFN-γ producing CD4^+^ T cells and CD8^+^ T cells in the lung of Sema3E-Fc treated mice compared to saline-Fc treated mice (**Figures 3**, **4**). Moreover, we found a more significant number of IL-17^+^ T cells in the lungs of Sema3E-Fc treated mice than saline-Fc treated mice after *Cm* infection (**Figures 5A, B**). Together, these data confirm that Sema3E can promote Th1/Tc1 and Th17 cell responses in the local tissues (lung) after *Cm* infection *in vivo.*

### Sema3E treatment reduced CD4^+^CD25^+^FoxP3^+^ regulatory T cells in the lung after *Cm* infection

**Figure 6:**
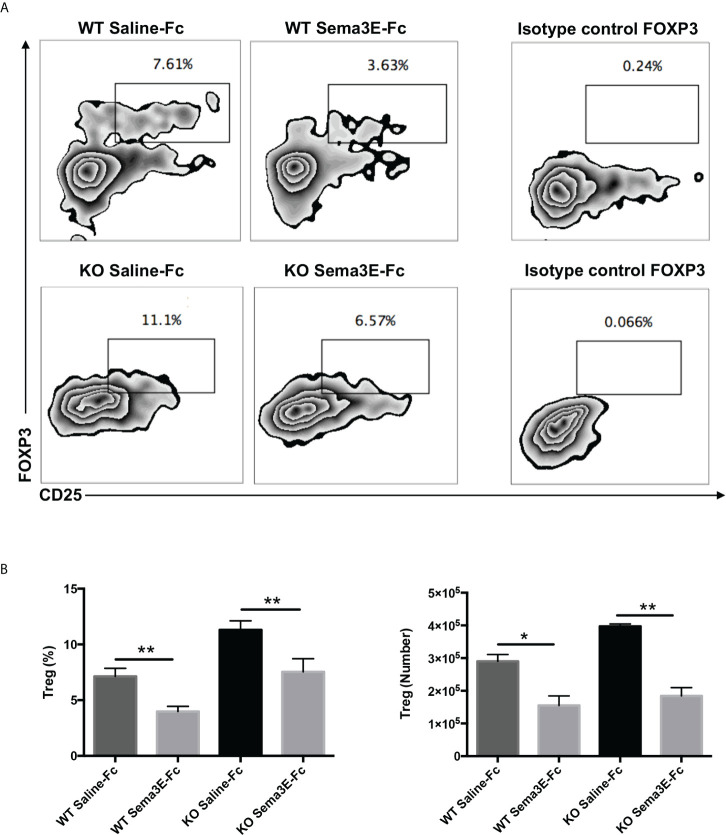
Lower Treg cells in the lungs of Sema3E-Fc treated mice following *Cm* infection. Mice were intranasally inoculated with 1x10^3^ IFUs of *Cm*. Lung cells were collected from Sema3E-Fc, or saline-Fc treated WT and Sema3E KO mice on day 7 post-infection and stained for expression of CD3, CD4, and CD25. FoxP3 intranuclear staining was done on T cells to analyze Treg cells as described in Materials and Methods. **(A)** Representative flow cytometric images of Treg cells in the lungs. **(B)** The percentages and number of Treg cells in the lung. Data are shown as mean ± SD (n = 3) and represent one of three independent experiments with similar results. *p < 0.05, **p < 0.01.

Since we observed lower IL‐10 levels in the spleen and lung of Sema3E-Fc treated mice and Treg is one of the primary sources of this cytokine, we next examined CD4^+^ CD25^+^ Foxp3^+^ T cells in the lungs of WT and Sema3E KO mice treated with Sema3E-Fc following *Cm* infection. We found that the proportion and number of CD4+CD25+ Foxp3+ T cells were significantly lower in Sema3E-Fc treated mice than saline-Fc treated mice in both Sema3E intact and deficient conditions (**Figures 6A, B**). The results suggest that Sema3E treatment can impede Treg responses during *Cm* infection.

### Sema3E treatment alters DC surface molecule expression and the development of DC subsets

**Figure 7:**
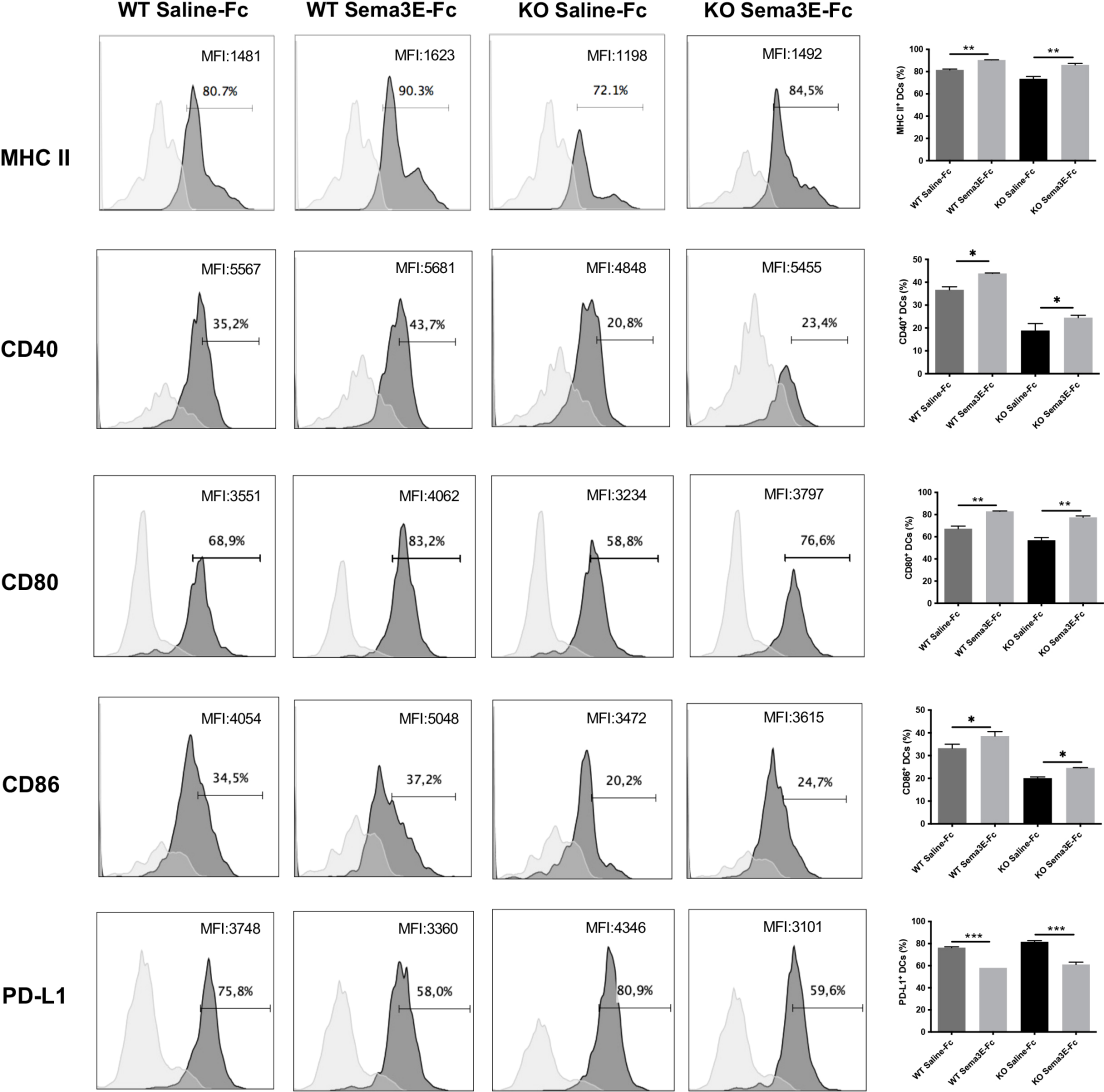
Sema3E treated mice showed the altered surface phenotype of DCs following *Cm* infection. Sema3E KO and WT mice were infected intranasally with *Cm*, treated with Sema3E-Fc, or saline-Fc, and sacrificed after 3 days. DCs were isolated from the lungs using CD11c microbeads, and cells were stained for surface markers and analyzed using flow cytometry. Expression of CD40, CD80, CD86 and PDL-1 on CD11c^+^ MHCII^+^ cells (dark shaded histograms) and isotype control (light shaded histograms) were shown. MHCII expression on CD11c^+^ cells and isotype control were recorded. The percentages and mean fluorescence intensity (MFI) of positive cells were indicated. One of the two independent experiments with similar results is shown (n = 3). *p < 0.05, **p < 0.01, ***p < 0.001.

We next evaluated whether the exogenous Sema3E treatment of WT and Sema3E KO mice affects the surface expression of co-stimulatory and inhibitory molecules on dendritic cells after chlamydial infection. Compared to saline-Fc treated mice, Sema3E-Fc treated WT, and Sema3E KO mice showed higher MHC‐II, CD40, CD80, and CD86 molecules on the surface of DCs following infection (**Figure 7**). In contrast, a lower percentage of PD-L1, an inhibitory surface molecule expressing DCs, was found in Sema3E-Fc treated mice compared to saline-Fc treated mice (**Figure 7**).

**Figure 8:**
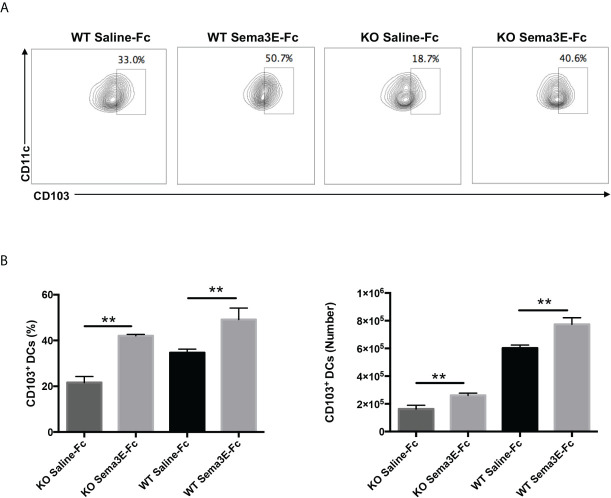
Higher CD103^+^ lung DC subset in Sema3E-Fc treated mice than saline-Fc treated mice after *Cm* infection. Mice were intranasally inoculated with 1x10^3^ IFUs of *Cm*. Lung cells were collected from Sema3E-Fc, or saline-Fc treated WT and Sema3E KO mice on day 3 post-infection, stained for surface markers and analyzed using flow cytometry. **(A)** Representative flow cytometric images of CD103^+^ lung DC subset. **(B)** The percentages and number of CD103^+^ lung DC subset. Data are shown as mean ± SD (n = 3) and represent one of three independent experiments with similar results. **p < 0.01.

Previous studies showed that different DC subsets exhibit variable capacity in inducing a protective immune response to *Cm* infection (13, 28). Notably, the CD103^+^ lung DC subset is reportedly more potent in inducing Th1/Th17 response to *Cm* infection than the CD11b^+^ lung DC subset (13). We, therefore, also analyzed CD103 expression in the lung DCs of Sema3E-treated mice. We found that Sema3E treatment increased the proportion and number of CD103^+^ DC subsets in the lung (**Figures 8A, B**). The results suggest that Sema3E plays a critical role in the recruitment and development of Th1/Th17 promoting DCs, including the CD103+ DC subset following chlamydial infection.

## Discussion

A large body of evidence shows that Sema3E is involved in regulating immune responses (19, 22, 25, 30). We recently reported that Sema3E is critical for host defence against bacterial infection, mainly based on a study to compare WT and Sema3E KO mice. In this study, we used a different approach, supplement of exogenous Sema3E, to further confirm the role of this molecule in host defence and explore the potential of Sema3E administration as prevention and therapeutic strategy for bacterial infections. We found that exogenous Sema3E treatment can significantly improve the condition of mice with intact or deficient Sema3E following chlamydial infection in the lung. The improvements are characterized by lower chlamydial growth, less severe lung pathology, and reduced body weight loss following infection. Interestingly, we found that the administration of exogenous Sema3E to either Sema3E KO or WT mice can modulate T cell responses, preferentially enhancing the right cytokine profile to fight against the infection. Specifically, we have observed that Sema3E treatment enhances IFN-γ and IL-17 production but reduces IL-10 and IL-4 cytokine response in the lungs and spleen after chlamydial infection. This observation was similar to what was shown in the study of allergic asthma, where Sema3E treatment enhanced the secretion of IFN-γ and reduced IL-4 in the airways upon house dust mite (HDM) challenge (26). The T cell-modulating effect of Sema3E appears related to its impact on DC development, including DC subsets. The changes are consistent with the increase of Sema3E levels in the exogenous Sema3E-treated mice (data not shown). These studies further confirm the promoting role of Sema3E for protective immunity against bacterial infection. More importantly, the finding that exogenous Sema3E can promote protective immunity in both Sema3E deficient and intact mice strongly suggests the potential of this protein as a target in developing novel preventive and therapeutic approaches against bacterial infections and other diseases.

The most significant observation in this study is the influence of Sema3E on DC development/recruitment. We observed that Sema3E treatment enhanced the frequency and numbers of CD103^+^ DC subset in the lung. The preferential protective role of the CD103^+^ pulmonary cDCs subset has been reported in chlamydial lung infection. Our previous studies using the adoptive transfer approach have shown that CD103^+^ pulmonary DCs induces more robust Th1 and Th17 responses than CD11b^+^ DCs (13). The finding in the present study concurs with the reported data, showing an association of increased CD103^+^ DCs with enhanced Th1/Th17 response in the Sema3E treated mice. Notably, the modulating function of Sema3E in lung DC subsets was recently reported in an allergic asthma model induced by HDM. The study found that CD103^+^ DCs from Sema3E treated mice induced higher IFN-γ production by T cells in an ex vivo DC-T co-culture system than DCs from saline-Fc treated mice (26). Future studies are needed to delineate the role of Sema3E in the preferential development of specific DC subset and consequent promotion of the types of T cell response in infections.

In addition to the enhanced CD103+ DC subset, we also found that Sema3E-Fc treated mice, compared to saline-Fc treated mice, exhibited higher surface expression of MHC‐II and the co-stimulatory CD40, CD80, and CD86 molecules but a lower expression of PD-L1, an inhibitory surface molecule. The maturation of DCs with higher expression of MHC and co-stimulatory molecules by DC is critical for developing protective immunity against chlamydial infection (31).

Another interesting finding in the present study is the inhibitory effect of Sema3E treatment on Treg responses in chlamydial infection. Treg plays a significant role in regulating immune response, which can be favourable and detrimental in host defence against infection. In *Chlamydia* studies, Treg is more associated with the inhibition of protective immunity. Several recent studies have shown the detrimental role of Treg cells in immunity to chlamydial infection. Depletion of Tregs reduced genital *Cm* infection by enhancing Th1 response (32). Inhibition of Treg response is one of the mechanisms by which NK cells protect against chlamydial lung infection (11, 33). In agreement with reduced Treg response, we also observed lower IL-10 cytokine in the lungs and spleen of Sema3E-treated mice. The inhibitory effect of Sema3E on PD-L1 expression on the surface of DCs is likely necessary for the suppression of Treg by Sema3E. In addition, the promoting effect of Sema3E on Th1 and Th17 responses can also participate in the inhibition of Treg. Indeed, it has been found that the expression of PD-L1 in DCs can inhibit Th1/Th17 responses, while Th17 is essential for the Th1-inducing DCs (13). Moreover, higher expression of PD-L1 in DC was also associated with lower IL-12 but more elevated IL-10 cytokines, thereby promoting Treg responses (11).

Chlamydial infections are routinely treated with antibiotics such as azithromycin or doxycycline (34). Considering that antibiotic treatments are associated with concerns such as side effects of treatment, recurrent infections, antibiotic resistance, and the safety in pregnancy (35–39), the development of non-antibiotic-based therapy is needed. Our studies showed that exogenous Sema3E treatment could be considered a novel therapeutic approach to treat chlamydial infections or be used as a supplement to mitigate the side effect of antibiotics. We have previously reported the endogenous secretion of Sema3E in response to chlamydial lung infection in mice (27). The present study suggests that higher levels of Sema3E would further enhance immunity to chlamydial infection *in vivo*. Enhancing protective immunity in both Sema3E deficient and intact mice by exogenous Sema3E treatment is particularly encouraging. It would be important to study the prevalence of Sema3E deficiency in humans and the relationship with chlamydial persistency in patients. Apart from chlamydial infection, accumulating evidence suggests the relevance of semaphorins as novel targets in cancer, autoimmune and allergic disorders. The therapeutic ability of the uncleavable variant of Sema3E (Uncl-Sema3E) was demonstrated in multiple tumour models where it acts as a novel inhibitor of tumour growth, metastasis, and angiogenesis (40). Also, *in vivo* studies on mice indicate that Sema3E treatment reduces allergic asthma by reducing eosinophilic inflammation, serum IgE level, and Th2 cytokine response and is proposed as a novel treatment option for allergic asthma (26). However, the efficacy of semaphorins has remained to be examined in clinical settings. Much more study in this area is needed.

In summary, using a model of administration of exogenous Sema3E, we confirmed the role of this molecule in host defence against chlamydial infection and revealed the potential of Sema3E treatment to restore the defect of Sema3E deficient individuals in immune protection and to enhance the protection in intact individuals. The study suggests that the immunomodulatory function of Sema3E on T cell and DC function may be considered in the development of preventive/therapeutic strategies in infectious/inflammatory diseases.

## Data availability statement

The original contributions presented in the study are included in the article/**Supplementary Material**. Further inquiries can be directed to the corresponding author.

## Ethics statement

The animal study was reviewed and approved by The University of Manitoba Animal Ethics Committee (Protocol # 19-029).

## Author contributions

RT, conceptualization, investigation, methodology, data analysis and writing. SW, investigation and methodology. RR and YP, investigation. AG, resources. XY, conceptualization, funding acquisition, resources, project administration, supervision, and writing.

## Funding

This work was supported by a grant from the Canadian Institutes of Health Research (MOP-130423) to XY, who was the Canada Research Chair in Infection and Immunity. RT is the recipient of the Research Manitoba graduate studentship.

## Conflict of interest

The authors declare that the research was conducted in the absence of any commercial or financial relationships that could be construed as a potential conflict of interest.

## Publisher’s note

All claims expressed in this article are solely those of the authors and do not necessarily represent those of their affiliated organizations, or those of the publisher, the editors and the reviewers. Any product that may be evaluated in this article, or claim that may be made by its manufacturer, is not guaranteed or endorsed by the publisher.

## Supplementary material

Supplementary Figure 1
**(A)** Exogenous recombinant Sema3E-Fc was administered to mice groups intranasally 2 hours before *Cm* infection and day 1 to day 6 after infection. **(B)** Gating strategy of lung DC. Lung DCs were identified according to CD11c^hi^MHC-II^hi^F4/80^-^ expression. **(C)** Gating strategy of lung T cells. Lymphocytes were identified based on surface expression of CD3 and further gated on CD4 and CD8.
