# Supplementary figures and images for: Exogenous Semaphorin 3E treatment protects against chlamydial lung infection in mice
Source: Front Immunol. 2022 Aug 2;13:882412. doi: 10.3389/fimmu.2022.882412 (PMC9379098; doi:10.3389/fimmu.2022.882412)

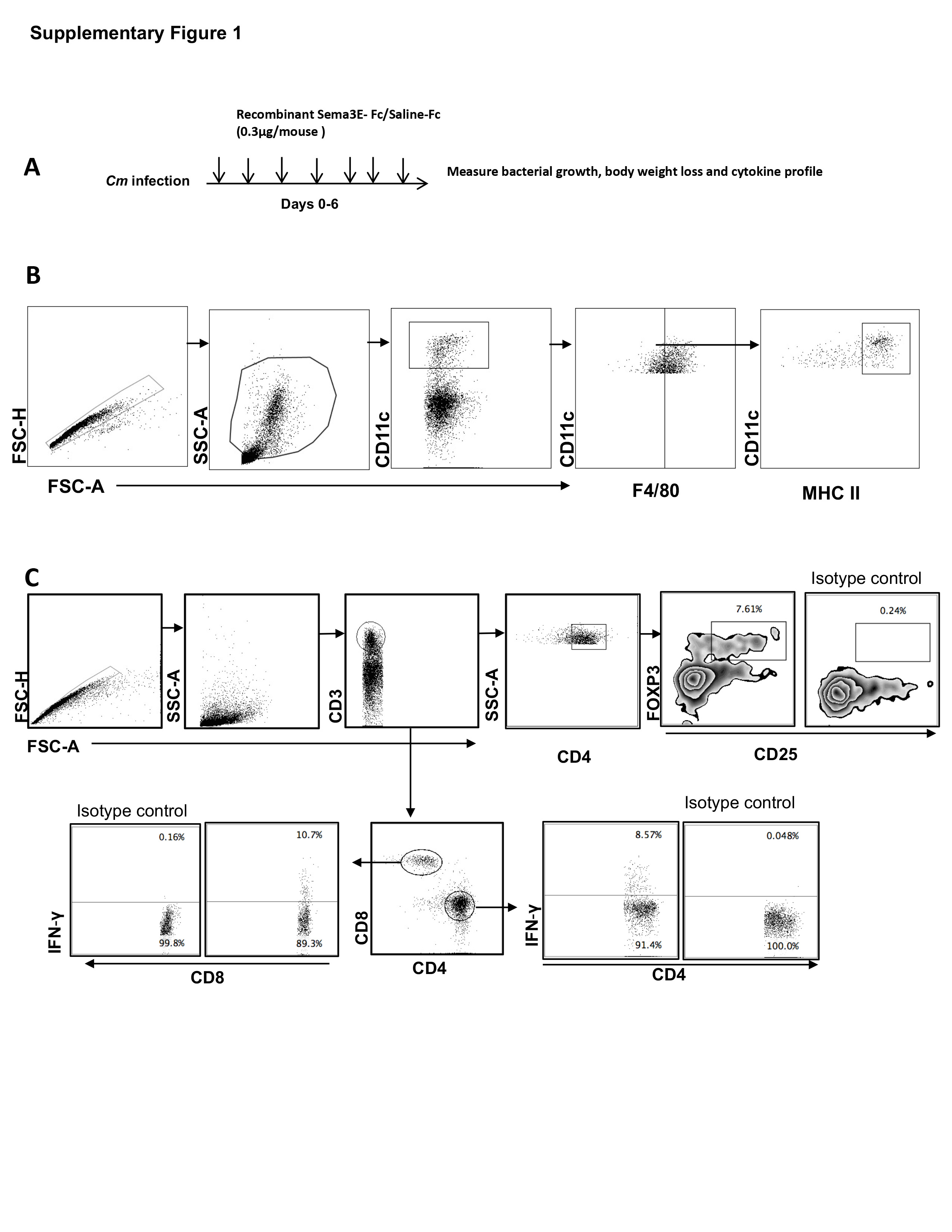

Supplement: Supplementary Figure 1 — (A) Exogenous recombinant Sema3E-Fc was administered to mice groups intranasally 2 hours before Cm infection and day 1 to day 6 after infection. (B) Gating strategy of lung DC. Lung DCs were identified according to CD11chiMHC-IIhiF4/80- expression. (C) Gating strategy of lung T cells. Lymphocytes were identified based on surface expression of CD3 and further gated on CD4 and CD8. [file Image_1.jpeg]
